# Synergistic Effects of Chitosan and Fish Oil on Lipid Metabolism in Rats Fed a High-Fat and Low-Carbohydrate Diet

**DOI:** 10.3390/nu16234080

**Published:** 2024-11-27

**Authors:** Shing-Hwa Liu, Ting-Yu Chang, Shih-Hou Liu, Meng-Tsan Chiang

**Affiliations:** 1 Graduate Institute of Toxicology, College of Medicine, National Taiwan University, Taipei 10051, Taiwan; shinghwaliu@ntu.edu.tw (S.-H.L.); d03447003@ntu.edu.tw (T.-Y.C.); 2 Department of Pediatrics, College of Medicine, National Taiwan University Hospital, Taipei 10041, Taiwan; 3 Department of Medical Research, China Medical University Hospital, China Medical University, Taichung 40402, Taiwan; 4 Department of Food Science, College of Life Science, National Taiwan Ocean University, Keelung 20224, Taiwan; 11132022@mail.ntou.edu.tw

**Keywords:** chitosan, fish oil, high-fat and low-carbohydrate diet, lipid metabolism

## Abstract

Background/Objectives: Although high-fat, low-carbohydrate diets are used for weight loss and type 2 diabetes management, their high-fat content may have negative effects. This study examines the effects of replacing cellulose with chitosan and part of the fat with fish oil in a high-fat, low-carbohydrate diet on lipid metabolism in rats. Methods: The experiment involved 35 six-week-old male SD rats, divided into five groups: normal control diet (ND), high-fat diet (HF), high-fat, low-carbohydrate diet (LC), LC with 5% chitosan (LC-CH), and LC with 5% chitosan and 5% fish oil (LC-CHF). Results: After 15 weeks, the HF group had the highest liver weight, and the LC group had the highest adipose tissue weight. The LC-CHF group showed significantly reduced body, liver, and adipose tissue weights, lower ALT, AST, TNF-α, and cholesterol levels, as well as improved liver enzyme activity and fat synthesis regulation. LC-CHF also promoted fat breakdown in adipose tissue, reducing adipocyte size. Conclusions: Our findings suggest the modified high-fat, low-carbohydrate diet with chitosan and fish oil improved obesity and fatty liver outcomes compared to a standard high-fat diet.

## 1. Introduction

The global increase in obesity and its associated metabolic disorders presents a pressing public health challenge, primarily driven by the widespread consumption of energy-dense, high-fat diets and sedentary behaviors [1]. Diet-induced obesity (DIO) is often accompanied by a constellation of metabolic dysfunctions, including dyslipidemia [2], hepatic steatosis [3], systemic inflammation [4], and oxidative stress [5], which significantly elevate the risk of non-communicable diseases such as cardiovascular disease [6], type 2 diabetes [7], and non-alcoholic fatty liver disease (NAFLD) [8]. As the prevalence of these conditions continues to grow, there is an urgent need for safe and effective nutritional interventions that can mitigate the adverse metabolic impacts of high-fat diets and support metabolic homeostasis.

Chitosan, a naturally occurring polysaccharide derived from the chitin in crustacean shells, has shown promise as a dietary supplement with lipid-lowering properties [9,10]. Its mechanism primarily involves binding to lipids in the gastrointestinal tract, thereby inhibiting their absorption and enhancing fecal excretion [11]. It has been shown to help lower LDL cholesterol by binding with fats and bile acids, encouraging the body to use more cholesterol to produce bile [11,12]. It has the unique ability to bind with dietary fats in the digestive tract, forming complexes that cannot be absorbed by the intestines. As a result, these fats are excreted rather than stored in the body, which helps reduce fat accumulation and calorie intake. Additionally, chitosan aids in cholesterol regulation by binding with bile acids, which are derived from cholesterol [11]. This forces the body to use more cholesterol to produce new bile acids, ultimately lowering the levels of LDL cholesterol in the bloodstream. Chitosan may also help improve lipid profiles by reducing LDL while potentially increasing HDL cholesterol, which supports cardiovascular health [13,14]. By limiting fat absorption and influencing cholesterol metabolism, chitosan contributes to better overall lipid management, making it beneficial for weight control and reducing the risk of metabolic disorders such as obesity and dyslipidemia. Similarly, fish oil, rich in omega-3 polyunsaturated fatty acids (PUFAs) such as eicosapentaenoic acid (EPA) and docosahexaenoic acid (DHA), exerts numerous beneficial effects on lipid metabolism, inflammation, and insulin sensitivity [15,16,17]. Omega-3 fatty acids are known to influence the expression of genes involved in lipid synthesis, β-oxidation, and inflammatory signaling, positioning fish oil as a complementary intervention in high-fat diet models [18,19]. Excessive accumulation of triglyceride (TG) in the liver can lead to NAFLD. Fish oil supplementation has been shown to improve hepatic steatosis in both humans and rodents by reducing the expression of SREBP1c, thereby inhibiting lipid synthesis-related genes such as FAS, ACC, and SCD1. Additionally, fish oil enhances PPARα activity, which promotes β-oxidation and further aids in reducing lipid accumulation in the liver [20].

This study investigated the synergistic effects of chitosan and fish oil in attenuating the metabolic consequences of a low-carbohydrate, high-fat (LC) diet, a common dietary model for inducing obesity and metabolic syndrome in experimental animals. We aim to determine whether the combined supplementation of chitosan and fish oil can mitigate the effects of a high-fat diet on lipid accumulation, and inflammatory response, and whether these agents can offer protective effects against diet-induced metabolic disturbances. The outcomes of this study may provide novel insights into the therapeutic potential of chitosan and fish oil as part of dietary strategies aimed at reducing the risk factors associated with high-fat diets. Understanding the molecular and physiological impacts of these supplements on metabolic processes will contribute to the development of targeted nutritional approaches for the prevention and management of obesity and its related comorbidities.

## 2. Materials and Methods

### 2.1. Experimental Animals, Diets, and Sample Collection

The animal experiments were approved by the Animal House Management Committee of the National Taiwan Ocean University and were conducted according to the guidelines for the care and use of laboratory animals. Thirty-five six-week-old Sprague Dawley (SD) rats were purchased from BioLASCO (Taipei, Taiwan). The animals were kept in cages within an animal room, where temperature and humidity were controlled, and a 12 h light/12 h dark cycle was maintained. During the acclimatization period, they were fed a solid diet (Laboratory Rodent Diet 5001, PMI Feed, Inc., St. Louis, MO, USA) for two weeks. After this period, they were randomly divided into five groups with seven rats per group. The groups were as follows: (1) ND: normal control diet (3% lard + 2% soybean oil) + 5% cellulose; (2) HF: high-fat diet (18% lard + 2% soybean oil) + 5% cellulose; (3) LC: low carbohydrate, high-fat diet (35% lard + 2% soybean oil) + 5% cellulose; (4) LC-CH: low carbohydrate, high-fat diet + 5% chitosan; and (5) LC-CHF: CH diet (30% lard + 2% soybean oil + 5% fish oil) + 5% chitosan (Table 1). Food and water were provided ad libitum. Body weight was measured weekly, and food intake was recorded three times per week. The experimental animals were fed according to their respective diet formulas for a total of 15 weeks. Feces were collected and weighed three days prior to sacrifice. A 12 h fasting period is required before sacrifice to standardize metabolic measurements and minimize variability caused by feeding status [21]. Blood, liver, and perirenal and para-epididymal adipose tissue samples were collected after the rats were euthanized under anesthesia with CO_2_/O_2_ (70:30) at the end of the experiment. Plasma was separated by centrifuging the blood at 3000 rpm for 20 min at 4 °C. All samples were immediately frozen and stored at −80 °C for later analysis.

### 2.2. Measurement of Total Cholesterol (TC), Triglyceride (TG), and Plasma Lipoprotein Cholesterol

To measure the total cholesterol in plasma, 10 μL of plasma was mixed with 1 mL of a cholesterol enzymatic reagent (#CH7945, Randox Laboratories Ltd., Crumlin, UK) in a tube. After thorough mixing, the reaction was carried out for 5 min in a water bath set at 37 °C (B206, FirstTek Scientific, New Taipei City, Taiwan). The absorbance was then measured at 500 nm using a spectrophotometer (UV/VIS-7800, JASCO International Co., Ltd., Tokyo, Japan). To measure triglyceride levels, 10 μL of plasma was combined with 1 mL of a triglyceride enzymatic reagent (#TR213, Randox Laboratories Ltd., Crumlin, UK) in a tube. The mixture was thoroughly shaken and then incubated in a 37 °C water bath (B206, FirstTek Scientific, New Taipei City, Taiwan) for 5 min. Absorbance was measured at 500 nm using a spectrophotometer (UV/VIS-7800, JASCO International Co., Ltd., Tokyo, Japan). For plasma lipoprotein analysis, a 110 μL plasma sample was mixed with KBr solutions of different densities (1.006 and 1.12 g/mL) and shaken well. Then, 200 μL of each mixed solution was centrifuged at 40,000 rpm for 3 h at 10 °C using an ultracentrifuge (SCP 85G, RPL42T Rotor, Hitachi, Tokyo, Japan). The supernatant and pellet were separated (100 μL from each layer) based on lipoprotein density. Cholesterol concentration in both layers was measured using an enzymatic kit, and HDL-C and LDL-C levels in plasma were calculated.

### 2.3. Measurement of Aspartate Aminotransferase (AST) and Alanine Aminotransferase (ALT) Activities

AST and ALT activities were assessed using RANDOX enzymatic kits (Antrim, UK) with absorbance readings taken at 340 nm. Measurements were performed kinetically with a Hitachi U-2800A spectrophotometer (Tokyo, Japan), following the manufacturer’s instructions.

### 2.4. Measurement of β-Hydroxybutyrate, TNF-α, and IL-6 Content

Rat β-hydroxybutyrate enzyme immunometric assay kit (R&D system, Inc., Minneapolis, MN, USA) was used to determine the level of β-hydroxybutyrate. The plasma sample was analyzed using a rat TNF-α enzyme immunometric assay kit (R&D Systems, Inc., Minneapolis, MN, USA). Absorbance was measured at 450 nm with an enzyme-linked immunosorbent assay reader (VersaMax microplate, Molecular Devices, Hampton, NH, USA), and the concentration was calculated by comparing the absorbance values with those of the standard. IL-6 levels were determined using a rat IL-6 enzyme immunometric assay kit (R&D system, Inc., Minneapolis, MN, USA).

### 2.5. Measurement of Hepatic Enzyme Activities: Acetyl-CoA Carboxylase (ACC), Fatty Acid Synthase (FAS), and HMG-CoA Reductase

The activities of ACC, FAS, and HMG-CoA reductase in the liver were measured by observing NADPH consumption, indicated by changes in absorbance at 340 nm. For ACC activity, acetyl-CoA, ATP, and bicarbonate were provided as substrates, while for FAS, acetyl-CoA and malonyl-CoA were used. HMG-CoA reductase activity was measured using HMG-CoA and NADPH+H⁺ as substrates in a cholesterol synthesis reaction. Each reaction mixture was prepared, added sequentially to a 96-well plate, and mixed using a rotary shaker. Absorbance changes were recorded at 37 °C using an immunosorbent assay reader (VersaMax, Molecular Devices, Hampton, NH, USA) over 5 min for ACC and FAS and 20 min for HMG-CoA reductase.

### 2.6. Hematoxylin and Eosin (H&E) Staining

Tissues were collected, fixed in 10% buffered formalin, embedded in paraffin, and sliced into 4 µm thick sections. Slides were removed from a −20 °C freezer and placed in a 55 °C oven for 15 min. Deparaffinization was performed by immersing the slides in xylene for 5 min twice, followed by rehydration with 100%, 90%, and 70% ethanol for 5 min each. The slides were stained with hematoxylin, rinsed under running water for 15 min, and then stained with eosin. They were washed until the water ran clear, then dehydrated sequentially in 70%, 90%, and 100% ethanol, followed by a final 5 min immersion in xylene. The distribution of fat vacuoles was then recorded using an upright fluorescence microscope (U-LH100HG, BX53, Olympus Co., Tokyo, Japan).

### 2.7. Fecal Lipid Extraction

The dried feces were ground into a powder using a grinder (HM-L14202BL, Sampo Inc., Taoyuan, Taiwan) and stored at −20 °C. For lipid extraction, a modified version of Folch’s method [22] was used. Add 0.2 g of fecal sample to a chloroform/methanol mixture (2:1, *v*/*v*) 20 times the fecal weight (4 mL) in a screw-cap tube. The mixture was agitated using a rotating tube mixer (T-101, FirsTek Scientific Co., Ltd., New Taipei City, Taiwan) for 72 h and then centrifuged at 3000 rpm for 10 min at 4 °C using a low-speed centrifuge (Hamic CF7D2, Hitachi, Tokyo, Japan). The supernatant containing the extracted fecal lipids was stored at −20 °C for further analysis. 

### 2.8. Statistical Analysis

Data are expressed as means ± S.D. Statistical differences between each experimental sample and its respective control were evaluated using one-way ANOVA, followed by Tukey’s post hoc test. A *p*-value of less than 0.05 was statistically significant. Statistical analysis was conducted with GraphPad Prism software, version 8.0 (Boston, MA, USA).

## 3. Results

### 3.1. Effects of Chitosan and Chitosan/Fish Oil on Body Weight, Food Intake, Food Efficiency, and Organ Weight in Low-Carbohydrate/High-Fat Diet-Fed Rats

After 15 weeks, rats fed various experimental diets showed differences in body weight, weight gain, food intake, and food efficiency (Table 2). These parameters were chosen to assess the overall impact of the diets on obesity-related outcomes and fat distribution, reflecting the ability of dietary interventions to modulate energy storage and adiposity. Initial body weights across groups were similar, ranging from 270.75 ± 29.63 g in the normal diet (ND) group to 287.92 ± 16.24 g in the low-carbohydrate high-fat diet with 5% chitosan (LC-CH) group. By the end of the study, the low-carbohydrate high-fat (LC) group had the highest final body weight (704.28 ± 50.42 g), while the low-carbohydrate high-fat with both chitosan and fish oil (LC-CHF) group had the lowest (572.04 ± 40.42 g). The LC group also showed the greatest body weight gain (420.73 ± 43.88 g), whereas the LC-CHF group had the least (285.83 ± 47.94 g). In terms of daily food intake, the high-fat (HF) group consumed the most (24.53 ± 2.25 g/day) compared to the LC, LC-CH, and LC-CHF groups. There was no significant difference in food efficiency between the ND and HF groups, while the LC group showed the highest food efficiency (20.36 ± 1.12%). These results showed the LC was most effective in promoting weight gain and food efficiency in high-fat diet-fed rats, while the addition of both chitosan and fish oil (LC-CHF) reduced body weight, weight gain, and food intake, suggesting that these additives may counteract some effects of a high-fat diet.

We further examined the liver and adipose tissue weights in all groups (Table 3). The HF group had the highest liver weight (35.54 ± 5.76 g), while the LC-CHF group had the lowest liver weight (25.38 ± 3.74 g), with relative liver weights following a similar pattern. The LC group exhibited the highest perirenal adipose weight (27.99 ± 9.05 g) and the highest relative perirenal adipose weight, while both the HF and LC-CHF groups had lower perirenal adipose weights. The LC group also had the greatest epididymal adipose weight (20.54 ± 5.14 g), whereas the LC-CHF group showed the lowest epididymal adipose weight (12.03 ± 1.89 g). Total adipose tissue weight was highest in the LC group (48.53 ± 11.72 g) and lowest in the LC-CHF group (28.32 ± 4.07 g), with relative total adipose tissue weight similarly highest in the LC group. These findings suggest that the LC significantly increased adipose tissue accumulation, while the addition of chitosan and fish oil in the LC-CHF appeared to reduce fat accumulation and liver weight, indicating potential benefits in moderating the effects of a high-fat diet.

### 3.2. Effects of Both Chitosan and Chitosan/Fish Oil on Lipid Profiles and Lipid-Related Metabolic Changes in HF-Diet-Fed Rats

We then assessed the effects of chitosan and chitosan/fish oil on lipid profiles, including plasma and hepatic total cholesterol, triglycerides, HDL-C, and LDL-C + VLDL-C in plasma and hepatic tissues. These markers are critical indicators of lipid metabolism and dyslipidemia. They were selected to evaluate the effectiveness of chitosan and fish oil in improving lipid balance, as both have been shown to influence cholesterol and triglyceride levels. As shown in Table 4, rats on the ND had a total cholesterol (TC) level of 98.17 ± 6.78 mg/dL, while those on the HF showed an increase to 110.62 ± 40.41 mg/dL. In contrast, the LC group had a slightly lower cholesterol level at 94.44 ± 16.41 mg/dL, and the LC-CH group showed a further decrease to 84.42 ± 19.98 mg/dL. The LC-CHF group had the lowest total cholesterol level, recorded at 54.12 ± 10.95 mg/dL, which was significantly lower than the levels in the ND, HF, and LC groups. The ND group’s HDL-C level was 46.55 ± 10.49 mg/dL, while the HF group had a reduced HDL-C level of 27.14 ± 7.85 mg/dL (*p* < 0.05). The LC-CHF group demonstrated the lowest HDL-C level, at 14.13 ± 6.07 mg/dL. The LDL-C + VLDL-C level was the highest in the HF group (83.48 ± 42.44 mg/dL), while the LC-CHF group was significantly lower than the HF group at 39.99 ± 12.07 mg/dL (*p* < 0.05), indicating an improvement in blood lipid profile. Triglyceride (TG) levels were significantly decreased in the HF group (51.20 ± 10.80 mg/dL) and the LC-CHF group (46.24 ± 15.50 mg/dL) compared to the ND group (79.02 ± 26.24 mg/dL). Overall, the LC-CHF, which combines chitosan and fish oil, appeared to improve lipid profiles the most, lowering total cholesterol and LDL-C + VLDL-C levels.

**Figure 1:**
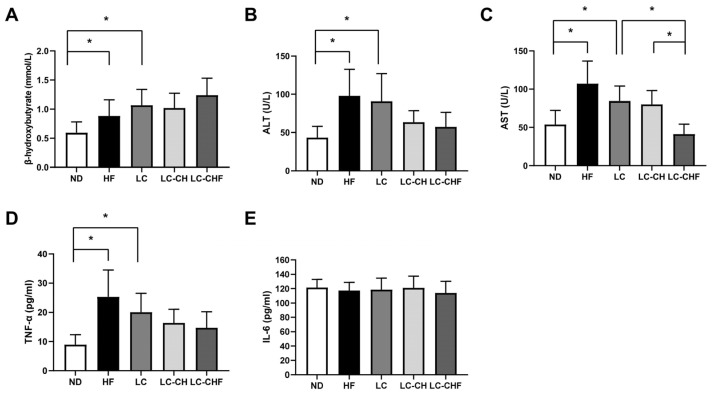
The result of plasma (**A**) β-hydroxybutyrate, (**B**) ALT, (**C**) AST, (**D**) TNF-α, and (**E**) IL-6 concentration in rats fed with the different experimental diets after 15 weeks. Results are expressed as the mean ± SD for each group (n = 7). The significant difference (*p* < 0.05) was analyzed by one-way ANOVA. * *p* ˂ 0.05 as compared between two indicated groups. ND: normal control diet; HF: high-fat diet; LC: low-carbohydrate + high-fat diet; LC-CH: low-carbohydrate + high-fat diet + 5% chitosan; and LC-CHF: low-carbohydrate + high-fat diet + 5% chitosan + 5% fish oil.

On the other hand, to demonstrate the effects of different dietary interventions on metabolic and inflammatory biomarkers in rats, we assessed the related plasma biomarkers. It showed that the HF and LC groups exhibited increased β-hydroxybutyrate levels (Figure 1A), suggesting altered ketone body metabolism. Additionally, liver enzymes such as alanine aminotransferase (ALT) and aspartate aminotransferase (AST), which serve as markers of liver function and stress, were significantly elevated in the HF and LC groups (Figure 1B,C), indicating hepatic stress. These markers allow for an assessment of the hepatic protective effects of chitosan and fish oil, particularly in mitigating the hepatotoxic effects of high-fat diets. We also examined the levels of inflammatory markers (TNF-α and IL-6), which were selected to assess systemic and hepatic inflammation, as both are known to play significant roles in the pathophysiology of obesity and NAFLD. TNF-α levels were also markedly higher in the HF and LC groups (Figure 1D), indicating an elevated inflammatory response. In contrast, the intervention groups, particularly LC-CHF, showed reduced AST levels compared to the LC and LC-CH groups, respectively (Figure 1C), suggesting a potential protective effect against liver stress. However, there were no significant differences in IL-6 levels across all groups (Figure 1E), indicating that the interventions did not markedly affect this inflammatory marker. These findings highlight that while a high-fat diet induces liver stress and inflammation, specific interventions might offer protective benefits for certain liver enzymes without significantly influencing IL-6 levels. Moreover, to explore whether dyslipidemia was linked to metabolic changes in the liver, the levels of hepatic total cholesterol and triglycerides and fat vacuoles were assessed as indicators of liver fat accumulation (hepatic steatosis). The HF group exhibited a substantial increase in both total cholesterol and triglyceride levels compared to the ND group, indicating heightened hepatic lipid accumulation with a high-fat diet (*p* < 0.05) (Table 5).

**Figure 2:**
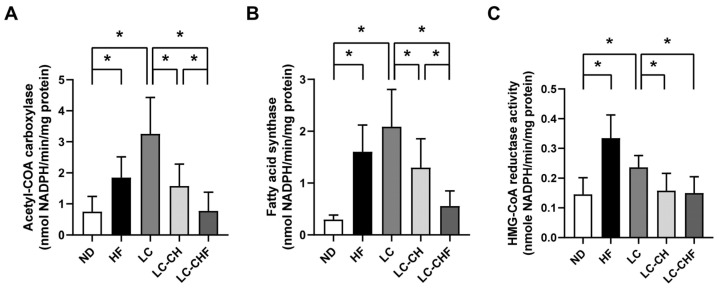
The results of hepatic enzyme activity of lipid biosynthesis in rats fed with the different experimental diets after 15 weeks. The activity of (**A**) acetyl-CoA carboxylase, (**B**) fatty acid synthase, and (**C**) HMG-CoA reductase in rats fed with different diets after a 15-week experiment. Results are expressed as the mean ± SD for each group (n = 7). The significant difference (*p* < 0.05) was analyzed by one-way ANOVA. * *p* ˂ 0.05 as compared between two indicated groups. ND: normal control diet; HF: high-fat diet; LC: low-carbohydrate + high-fat diet; LC-CH: low-carbohydrate + high-fat diet + 5% chitosan; and LC-CHF: low-carbohydrate + high-fat diet + 5% chitosan + 5% fish oil.

In contrast, the LC-CH and LC-CHF groups showed significantly lower total cholesterol levels compared to the HF and LC groups (*p* < 0.05). Elevated triglyceride levels in the LC group were effectively reversed in the LC-CHF group but not in the LC-CH group. These reductions suggest that the addition of chitosan and fish oil to a low-carbohydrate, high-fat diet may help mitigate hepatic lipid accumulation. We further examined the effects of different diets on enzyme activities related to lipid metabolism (ACC, FAS, and HMG-CoA reductase), which were analyzed to investigate how the interventions affect lipid synthesis and breakdown at a molecular level, providing insights into the mechanisms of lipid regulation in rats after 15 weeks. The HF and LC groups showed a significant increase in acetyl-CoA carboxylase, fatty acid synthase, and HMG-CoA reductase activities compared to the ND group (Figure 2A–C), indicating the enhanced lipid and cholesterol synthesis under a high-fat diet. However, the LC-CH and LC-CHF groups, which included chitosan and fish oil supplementation, exhibited significantly lower activities of these enzymes compared to the LC group. This reduction suggests that chitosan and fish oil may mitigate the effects of a high-fat diet on lipid synthesis by lowering the activity of key enzymes involved in lipid and cholesterol metabolism.

**Figure 3:**
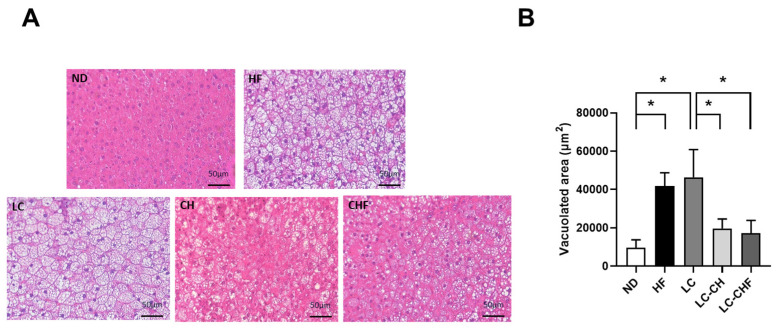
The results of hepatic morphology in rats fed with the different experimental diets after 15 weeks. Representative hematoxylin and eosin (H&E) stained (**A**) images of fat vacuoles in liver tissues were shown and (**B**) quantified. Results are expressed as the mean ± SD for each group (n = 7). The significant difference (*p* < 0.05) was analyzed by one-way ANOVA. * *p* ˂ 0.05 as compared between two indicated groups. ND: normal control diet; HF: high-fat diet; LC: low-carbohydrate + high-fat diet; LC-CH: low-carbohydrate + high-fat diet + 5% chitosan; and LC-CHF: low-carbohydrate + high-fat diet + 5% chitosan + 5% fish oil.

Histological analysis of liver tissue showed notable differences in lipid accumulation among the groups (Figure 3A). The HF and LC groups exhibited larger, more numerous vacuoles in liver cells, indicating increased hepatic lipid storage compared to the ND group. In contrast, both the LC-CH and LC-CHF groups displayed smaller and fewer vacuoles, implying that chitosan and fish oil may help reduce liver fat accumulation. The quantification of vacuolated areas supported these observations, with the HF and LC groups showing significantly larger vacuolated areas than the ND group (Figure 3B). However, the LC-CH and LC-CHF groups had significantly reduced vacuolated areas compared to the LC group, reinforcing the potential protective effect of chitosan and fish oil against hepatic lipid accumulation. These findings suggest that a high-fat diet promotes liver steatosis, while the inclusion of chitosan and fish oil may mitigate these effects.

### 3.3. Effects of Both Chitosan and Chitosan/Fish Oil on Adipose Tissue and Fecal Lipids in HF-Diet-Fed Rats

**Figure 4:**
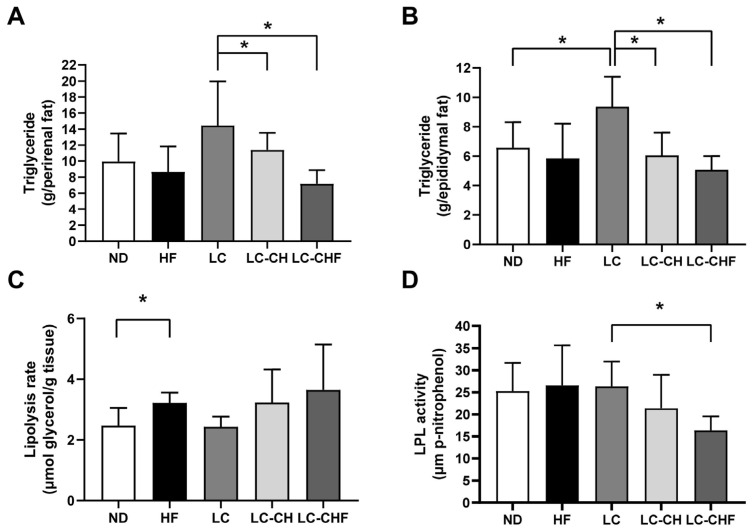
The results of (**A**) perirenal and (**B**) epididymal adipose tissue triglyceride concentration, and the change in (**C**) perirenal adipose tissue lipolysis rate and (**D**) lipoprotein lipase (LPL) activity in rats fed with the different experimental diets after 15 weeks. Results are expressed as the mean ± SD for each group (n = 7). The significant difference (*p* < 0.05) was analyzed by one-way ANOVA. * *p* ˂ 0.05 as compared between two indicated groups. ND: normal control diet; HF: high-fat diet; LC: low-carbohydrate + high-fat diet; LC-CH: low-carbohydrate + high-fat diet + 5% chitosan; and LC-CHF: low-carbohydrate + high-fat diet + 5% chitosan + 5% fish oil.

The LC led to higher triglyceride levels in both perirenal and epididymal fat compared to the ND and HF groups, although this was not statistically significant (Figure 4A,B). It indicated that a low-carbohydrate, high-fat diet promoted lipid accumulation in fat stores. However, supplementation with chitosan and chitosan/fish oil in the LC-CH and LC-CHF groups resulted in significantly lower triglyceride levels in these fat depots compared to the LC group (Figure 4A,B), suggesting that these supplements help reduce lipid accumulation. While the HF group showed a significantly higher lipolysis rate than the ND group (Figure 4C), there were no notable differences in lipolysis rate among the LC, LC-CH, and LC-CHF groups, indicating that chitosan and fish oil may not strongly influence fat breakdown. Interestingly, the LC-CHF group displayed significantly lower lipoprotein lipase (LPL) activity compared to the LC group (Figure 4D), which could limit triglyceride uptake in adipose tissue and contribute to reduced fat storage. Overall, these findings suggest that chitosan and fish oil supplementation may help counteract the fat accumulation associated with a low-carbohydrate, high-fat diet by reducing triglyceride storage in adipose tissue, potentially through decreased LPL activity.

**Figure 5:**
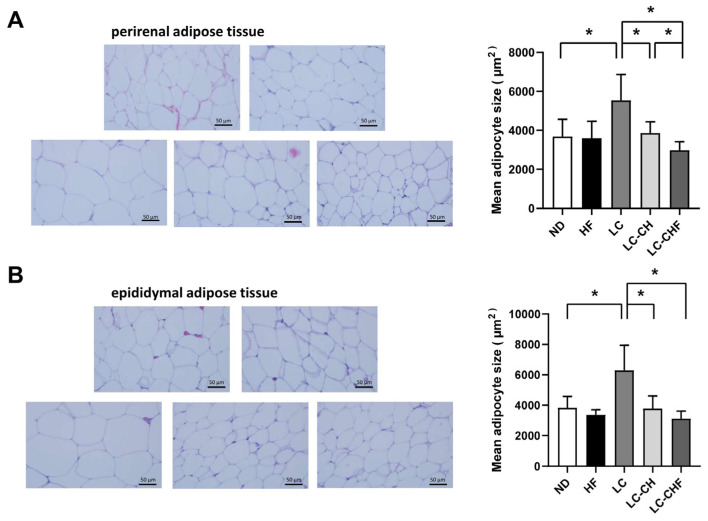
The results of perirenal adipose tissue morphology in rats fed with the different experimental diets after 15 weeks. (**A**) Histological morphology of adipocytes and (**B**) the mean adipose size. Results are expressed as the mean ± SD for each group (n = 7). The significant difference (*p* < 0.05) was analyzed by one-way ANOVA. * *p* ˂ 0.05 as compared between two indicated groups. ND: normal control diet; HF: high-fat diet; LC: low-carbohydrate + high-fat diet; LC-CH: low-carbohydrate + high-fat diet + 5% chitosan; and LC-CHF: low-carbohydrate + high-fat diet + 5% chitosan + 5% fish oil.

Moreover, in perirenal adipose tissue, the LC group exhibited significantly larger adipocytes compared to the ND groups (Figure 5A), indicating that a low-carbohydrate, high-fat diet promotes adipocyte hypertrophy in this fat depot. However, supplementation with chitosan and fish oil in the LC-CH and LC-CHF groups led to significantly smaller adipocytes compared to the LC group (Figure 5A), suggesting a mitigating effect on adipocyte enlargement. Similarly, in epididymal adipose tissue, the LC group showed notably larger adipocytes than the ND groups (Figure 5B). The LC-CH and LC-CHF groups again demonstrated significantly reduced adipocyte sizes compared to the LC group (Figure 5B), indicating that chitosan and fish oil supplementation may counteract the hypertrophic effects of adipocytes in a high-fat, low-carbohydrate diet-fed rats. These findings highlight the potential protective role of chitosan and fish oil in reducing adipocyte hypertrophy induced by a high-fat diet, potentially aiding in better fat storage management in both perirenal and epididymal adipose tissues.

We further examined the fecal weight and the levels of total cholesterol and triglycerides in the feces of rats after 15 weeks on different diets. As shown in Table 6, the LC group exhibited significantly higher fecal wet and dry weights compared to the ND groups, suggesting the increased fecal output on a low-carbohydrate, high-fat diet. Total cholesterol levels in feces, both in mg/g and mg/day, were significantly elevated in the HF and LC groups compared to the ND group. Notably, the LC, LC-CH, and LC-CHF groups had even higher fecal cholesterol levels than the HF group (*p* < 0.05), indicating that a low-carbohydrate, high-fat diet may enhance cholesterol excretion. Fecal cholesterol content in the LC-CHF group was significantly higher than that in the LC and LC-CH groups. For triglycerides, the LC group showed significantly higher fecal triglyceride levels compared to the ND group. However, there were no significant differences among the other groups. These results indicate that while a high-fat, low-carbohydrate diet increases fecal cholesterol and triglyceride levels, the addition of chitosan and fish oil can further enhance cholesterol elimination in feces. This suggests a beneficial role of chitosan and fish oil in managing lipid excretion in diets with high-fat and low-carbohydrates.

## 4. Discussion

The findings of this study provided important insights into the metabolic effects of chitosan and fish oil supplementation in the context of a high-fat, low-carbohydrate diet. Our results demonstrated that the combined supplementation of chitosan and fish oil (LC-CHF) significantly mitigated weight gain, reduced adiposity, and improved lipid profiles, suggesting a synergistic effect that addressed the key metabolic dysfunctions associated with high-fat diets.

The dosages of chitosan (5%) and fish oil (5%) were selected based on previous studies demonstrating their efficacy in improving lipid metabolism, reducing adiposity, and enhancing overall metabolic health in experimental models [23]. For chitosan, 5% has been shown to effectively bind dietary fats, enhance fecal lipid excretion, and reduce lipid absorption without adverse effects [24,25]. For fish oil, 5% provides an adequate supply of omega-3 polyunsaturated fatty acids (PUFAs), such as EPA and DHA, which promote β-oxidation, reduce inflammation, and suppress lipogenic pathways like SREBP1c and ACC [26,27,28]. The rationale for investigating the combined effects of chitosan and fish oil lies in their complementary mechanisms of action. Chitosan primarily targets dietary lipid absorption, while fish oil modulates systemic lipid metabolism and reduces inflammation. Together, these mechanisms are hypothesized to act synergistically by targeting multiple aspects of lipid metabolism. Chitosan enhances cholesterol and triglyceride excretion, while fish oil activates PPARα to promote lipid oxidation and suppress lipogenesis, resulting in improved lipid profiles, reduced adiposity, and mitigated hepatic lipid accumulation. This combination was designed to provide a more comprehensive strategy for managing diet-induced metabolic dysfunction compared to either component alone.

Firstly, the LC-CHF group showed the lowest final body weight, weight gain, and food intake compared to other high-fat diet groups. These findings suggest that chitosan and fish oil may counteract high-fat diet-induced obesity, potentially by enhancing lipid excretion and promoting lipid metabolism. The observed increase in fecal cholesterol levels supported the role of chitosan in binding lipids in the gastrointestinal tract, thereby reducing lipid absorption and enhancing excretion. This effect aligned with previous studies reporting that chitosan improved lipid profiles by promoting cholesterol and triglyceride elimination [23,29]. Furthermore, fish oil supplementation in the LC-CHF group was associated with improved plasma lipid profiles, including reduced total cholesterol, LDL-C, and VLDL-C levels. This improvement is likely due to the omega-3 fatty acid in fish oil, which has been shown to reduce hepatic triglyceride synthesis and enhance lipid oxidation. Our results are consistent with earlier findings that omega-3 fatty acids lower triglyceride and cholesterol levels, reduce hepatic VLDL production, and increase β-oxidation in adipose tissue [26]. Notably, the combined supplementation of chitosan and fish oil also led to reductions in lipid accumulation and hepatic steatosis, evidenced by smaller and fewer hepatic fat vacuoles and reduced vacuolated areas in histological analyses.

Despite the reduction in food intake, several findings suggest that chitosan and fish oil exert effects beyond caloric restriction. The LC, LC-CH, and LC-CHF groups exhibited significantly higher fecal cholesterol levels compared to the HF groups (Table 6), demonstrating chitosan’s ability to bind and enhance cholesterol excretion, independent of energy intake. Additionally, the improved plasma lipid profiles in the LC-CHF group, including lower LDL-C + VLDL-C and total cholesterol levels, align with the known lipid-modulating effects of omega-3 fatty acids in fish oil, which activate PPARα and suppress SREBP1c activity, directly influencing lipid metabolism [30,31]. Furthermore, the LC-CHF group showed marked suppression of lipogenic enzymes (ACC and FAS) (Figure 2A,B), suggesting a direct impact on hepatic lipid metabolism. While we acknowledge that the reduced food intake in the LC-CHF group likely contributed to the observed benefits, these findings collectively support the hypothesis that chitosan and fish oil exert direct metabolic effects that significantly improve lipid metabolism and oxidative stress. The reduction in adipose tissue size and lipid storage in the LC-CHF group suggests an attenuation of adipocyte hypertrophy. This effect may be attributed to the activation of PPARα and suppression of LXRα. The activation of PPARα has been linked to enhanced lipid oxidation and thermogenesis, while the suppression of LXRα can reduce lipogenesis, thus improving lipid metabolism and reducing adiposity [32,33,34]. In the present study, our findings align with previous work that chitosan and fish oil could modulate these signaling pathways, leading to reduced adipocyte size and decreased lipid storage in adipose tissues [35].

Chitosan primarily acts by binding dietary lipids in the gastrointestinal tract, reducing their absorption and enhancing fecal excretion. This effect is evident from the increased fecal cholesterol levels in the LC-CHF group. Chitosan has also been shown to influence lipid metabolism at the molecular level. It increases the expression of genes such as APOE and MTTP, which are involved in lipid transport, and activates AMPK, a central regulator of energy metabolism. AMPK activation suppresses downstream lipogenic pathways by inhibiting ACC and FAS, leading to reduced lipid synthesis and storage. Furthermore, chitosan promotes lipolysis in adipose tissues by enhancing the activity of hormone-sensitive lipase (HSL), contributing to reduced adiposity. Fish oil is rich in omega-3 polyunsaturated fatty acids (PUFAs), such as eicosapentaenoic acid (EPA) and docosahexaenoic acid (DHA), which act through several pathways. Omega-3 PUFAs are potent activators of PPARα, a nuclear receptor that upregulates genes involved in fatty acid β-oxidation, reducing lipid accumulation in the liver and adipose tissues. Fish oil also suppresses SREBP1c, a transcription factor that regulates lipogenic enzymes such as FAS and ACC, thereby reducing lipid synthesis. Additionally, omega-3 PUFAs modulate cholesterol metabolism by inhibiting HMG-CoA reductase activity, leading to decreased endogenous cholesterol synthesis and improved plasma lipid profiles.

The observed effects of chitosan and fish oil supplementation can be attributed to several molecular pathways regulating lipid metabolism, inflammation, and oxidative stress. One key mechanism involves the activation of PPARα, a nuclear receptor that promotes fatty acid β-oxidation and energy expenditure. The activation of PPARα likely contributed to the reduction in hepatic triglycerides and total cholesterol levels in the LC-CHF group, as enhanced β-oxidation reduces lipid storage in the liver and adipose tissues. Previous studies have shown that omega-3 fatty acids in fish oil are potent activators of PPARα, which may explain the improved lipid profiles and reduced adiposity observed in this study [36]. Simultaneously, the suppression of SREBP1c and LXRα in the LC-CHF group may have inhibited lipogenesis. These transcription factors are critical regulators of lipid synthesis, with SREBP1c controlling the expression of enzymes such as FAS and ACC [37]. The downregulation of SREBP1c and its downstream targets in the LC-CHF group aligns with the reductions in FAS and ACC activity observed in our data, further supporting the role of chitosan and fish oil in reducing lipogenesis and lipid accumulation in the liver and adipose tissues. Another notable pathway involves the inhibition of HMG-CoA reductase, a key enzyme in cholesterol biosynthesis. The decreased activity of HMG-CoA reductase in the LC-CHF group suggests that chitosan and fish oil supplementation may reduce endogenous cholesterol production, contributing to the observed improvements in plasma lipid profiles. The reduced LDL-C and VLDL-C levels in the LC-CHF group are consistent with this mechanism. In summary, the effects of chitosan and fish oil on lipid metabolism and inflammation appear to be mediated through the activation of PPARα, suppression of SREBP1c and LXRα, and inhibition of HMG-CoA reductase. These pathways collectively reduce lipogenesis, enhance lipid oxidation, and improve cholesterol metabolism. Linking these mechanisms to the observed reductions in adiposity and hepatic lipid accumulation provides a comprehensive understanding of the physiological implications of these dietary interventions.

The absence of a fish oil-only group was a limitation of the current study. Including a fish oil-only group would indeed provide a more comprehensive understanding of the specific contributions of fish oil and allow for a more accurate evaluation of the potential synergistic effects when combined with chitosan. Despite this limitation, the current study provides valuable insights into the effects of fish oil. Previous literature has extensively documented the independent effects of fish oil on lipid metabolism, including its ability to enhance PPARα activation, reduce inflammation, and improve lipid profiles [36]. These findings, combined with the current results, suggest complementary roles for chitosan and fish oil in targeting different aspects of lipid metabolism. Nevertheless, the inclusion of fish oil-only groups in future studies will increase the validity and depth of conclusions regarding synergy.

This study presents several novel insights into the synergistic effects of chitosan and fish oil on lipid metabolism and metabolic health in the context of a high-fat, low-carbohydrate diet. While previous research has examined the individual effects of chitosan or fish oil, this study uniquely evaluates their combined supplementation, providing evidence of their complementary mechanisms in improving lipid profiles, reducing adiposity, and mitigating hepatic lipid accumulation. One of the key contributions of this study is the comprehensive assessment of multiple metabolic parameters, including plasma lipid profiles, hepatic enzyme activities, and histological analyses of adipose tissue and liver. This multi-faceted approach offers a deeper understanding of how chitosan and fish oil jointly modulate lipid metabolism and inflammation, beyond what has been reported in earlier studies focusing on single interventions. Moreover, the study extends prior research by exploring the effects of chitosan and fish oil in a low-carbohydrate, high-fat dietary model, which better reflects certain modern dietary patterns. This distinction adds relevance to the findings, offering practical implications for dietary interventions in populations consuming similar diets. In summary, the novelty of this study lies in its focus on the synergistic effects of chitosan and fish oil, its comprehensive evaluation of metabolic outcomes, and its exploration of molecular mechanisms underlying these effects. By addressing these gaps in the literature, this research provides valuable insights that advance our understanding of dietary strategies for managing obesity and metabolic disorders.

Overall, our study suggests that the combination of chitosan and fish oil offers substantial benefits in counteracting the effects of a high-fat, low-carbohydrate diet. This combination effectively lowers body weight, improves lipid profiles, and reduces adiposity, making it a promising dietary intervention for managing obesity and metabolic disorders. Future studies should further explore the underlying mechanisms, particularly the role of chitosan and fish oil in gene expression related to lipid metabolism and inflammation, to better understand their potential in therapeutic applications for metabolic health.

## 5. Conclusions

This study showed that adding chitosan and fish oil to a high-fat, low-carbohydrate diet (LC-CHF) effectively reduced body weight gain, adiposity, and liver fat accumulation while improving lipid profiles. The LC-CHF lowered total cholesterol, LDL-C, and VLDL-C levels, enhanced cholesterol excretion, and decreased markers of liver stress, suggesting protective effects on lipid metabolism and hepatic health. These findings indicate that chitosan and fish oil supplementation may counteract the adverse effects of high-fat diets, offering a potential dietary approach for managing obesity and metabolic health. Future research should focus on elucidating the molecular mechanisms underlying these effects, particularly the roles of chitosan and fish oil in regulating lipid metabolism pathways and inflammatory responses. Additionally, long-term studies are needed to assess the sustainability of these benefits and their potential translation to human dietary interventions. Exploring variations in dosage and timing of supplementation could further optimize their therapeutic applications.

## Figures and Tables

**Table 1:** Experimental diet composition.

Ingredients (%)	ND	HF	LC	LC-CH	LC-CHF
Casein	20	20	20	20	20
Lard	3	18	35	35	30
Soybean oil	2	2	2	2	2
Fish oil					5
Vitamin mixture ^1^	1	1	1	1	1
Salt mixture ^2^	4	4	4	4	4
Cholesterol		0.5	0.5	0.5	0.5
Choline chloride	0.2	0.2	0.2	0.2	0.2
Cholic acid		0.2	0.2	0.2	0.2
Corn starch	64.8	49.1	32.1	32.1	32.1
Cellulose	5	5	5		
Chitosan				5	5
Total calories (kcal/100 g)	394.2	470.9	555.9	555.9	555.9
Carbohydrate (%)	68.29	43.83	24.89	24.89	24.89
Protein (%)	20.29	16.98	14.39	14.39	14.39
Fat (%)	11.41	39.18	60.71	60.71	60.71
Total (%)	100	100	100	100	100

ND: normal control diet (3% lard + 2% soybean oil) + 5% cellulose; HF: high-fat diet (18% lard +2% soybean oil) + 5% cellulose; LC: low-carbohydrate, high-fat diet (lard 35% + soybean oil 2%) + 5% cellulose; LC-CH: low-carbohydrate, high-fat diet + 5% chitosan; and LC-CHF: CH diet (lard 30% + soybean oil 2% + fish oil 5%) + 5% chitosan. ^1^ AIN-93 vitamin mixture; ^2^ AIN-93 mineral mixture.

**Table 2:** The results of body weight and food intake in rats fed with the different experimental diets after 15 weeks.

	ND	HF	LC	LC-CH	LC-CHF
Initial body weight (g)	270.75 ± 29.63	283.84 ± 24.74	283.55 ± 24.49	287.92 ± 16.24	286.21 ± 18.67
Final body weight (g)	616.15 ± 71.71	627.93 ± 52.73	704.28 ± 50.42 *^#^	613.97 ± 50.08 ^+^	572.04 ± 40.42 ^+^
Body weight gain (g)	345.39 ± 65.29	344.09 ± 54.02	420.73 ± 43.88	326.05 ± 60.70 ^+^	285.83 ± 47.94 ^+^
Food intake (g/day)	27.32 ± 2.17	24.53 ± 2.25	20.63 ± 1.25 *^#^	19.37 ± 1.91 *^#^	17.95 ± 1.28 *^#^
Food efficiency ^1^	12.56 ± 1.61	13.98 ± 1.35	20.36 ± 1.12 *^#^	16.72 ± 1.57 *^#+^	15.85 ± 1.67 *^+^

Results are expressed as the mean ± SD for each group (n = 7). The significant difference (*p* < 0.05) was analyzed by one-way ANOVA. * *p* ˂ 0.05 as compared with the ND group. ^#^ *p* ˂ 0.05 as compared with the HF group. ^+^ *p* ˂ 0.05 as compared with the LC group. ND: normal control diet; HF: high-fat diet; LC: low-carbohydrate + high-fat diet; LC-CH: low-carbohydrate + high-fat diet + 5% chitosan; and LC-CHF: low-carbohydrate + high-fat diet + 5% chitosan + 5% fish oil. ^1^ Food efficiency = [body weight gain (g) ÷ food intake (g/day)].

**Table 3:** The results of organ weight in rats fed with different experimental diets after 15 weeks.

	ND	HF	LC	LC-CH	LC-CHF
Liver weight (g)	18.18 ± 3.77	35.54 ± 5.76 *	34.30 ± 3.09 *	28.90 ± 4.84 *	25.38 ± 3.74 *^#+^
Relative liver weight (g/100 g BW)	2.93 ± 0.35	5.63 ± 0.49 *	4.87 ± 0.33 *^#^	4.69 ± 0.49 *^#^	4.42 ± 0.45 *^#^
Perirenal adipose weight (g)	20.71 ± 6.80	16.30 ± 4.55	27.99 ± 9.05 ^#^	22.75 ± 4.31	16.29 ± 2.77 ^+^
Relative perirenal adipose weight (g/100 g BW)	3.33 ± 0.83	2.58 ± 0.58	3.95 ± 1.12 ^#^	3.71 ± 0.66	2.83 ± 0.30
Epididymal adipose weight (g)	13.85 ± 2.43	12.45 ± 2.69	20.54 ± 5.14 *^#^	14.83 ± 3.03 ^+^	12.03 ± 1.89 ^+^
Relative epididymal adipose weight (g/100 g BW)	2.27 ± 0.44	1.98 ± 0.42	2.89 ± 0.55 ^#^	2.41 ± 0.40	2.10 ± 0.29 ^+^
Total adipose tissue weight (g)	34.56 ± 8.11	28.76 ± 6.45	48.53 ± 11.72 *^#^	37.59 ± 6.07	28.32 ± 4.07 ^+^
Relative total adipose tissue weight (g/100 g BW)	5.60 ± 1.04	4.56 ± 0.83	6.84 ± 1.30 ^#^	6.12 ± 0.80 ^#^	4.94 ± 0.44 ^+^

Results are expressed as the mean ± SD for each group (n = 7). The significant difference (*p* < 0.05) was analyzed by one-way ANOVA. * *p* ˂ 0.05 as compared with the ND group. ^#^ *p* ˂ 0.05 as compared with the HF group. ^+^ *p* ˂ 0.05 as compared with the LC group. ND: normal control diet; HF: high-fat diet; LC: low-carbohydrate + high-fat diet; LC-CH: low-carbohydrate + high-fat diet + 5% chitosan; and LC-CHF: low-carbohydrate + high-fat diet + 5% chitosan + 5% fish oil.

**Table 4:** The results of plasma lipids in rats fed with different experimental diets after 15 weeks.

(mg/dL)	ND	HF	LC	LC-CH	LC-CHF
Total cholesterol	98.17 ± 6.78	110.62 ± 40.41	94.44 ± 16.41	84.42 ± 19.98	54.12 ± 10.95 *^#+^
HDL-C	46.55 ± 10.49	27.14 ± 7.85 *	29.34 ± 11.26 *	26.92 ± 12.71 *	14.13 ± 6.07 *
LDL-C + VLDL-C	51.62 ± 14.67	83.48 ± 42.44	65.10 ± 19.76	57.50 ± 23.08	39.99 ± 12.07 ^#^
TC/HDL-C	2.21 ± 0.56	4.65 ± 3.00	3.70 ± 1.76	3.78 ± 1.92	4.66 ± 2.36
HDL-C/(LDL-C + VLDL-C)	1.04 ± 0.59	0.42 ± 0.24	0.51 ± 0.31	0.63 ± 0.61	0.41 ± 0.26
Triglyceride	79.02 ± 26.24	51.20 ± 10.80 *	52.05 ± 12.21	58.94 ± 17.87	46.24 ± 15.50 *

Results are expressed as the mean ± SD for each group (n = 7). The significant difference (*p* < 0.05) was analyzed by one-way ANOVA. * *p* ˂ 0.05 as compared with the ND group. ^#^ *p* ˂ 0.05 as compared with the HF group. ^+^ *p* ˂ 0.05 as compared with the LC group. ND: normal control diet; HF: high-fat diet; LC: low-carbohydrate + high-fat diet; LC-CH: low-carbohydrate + high-fat diet + 5% chitosan; and LC-CHF: low-carbohydrate + high-fat diet + 5% chitosan + 5% fish oil.

**Table 5:** The results of liver lipids in rats fed with different experimental diets after 15 weeks.

	ND	HF	LC	LC-CH	LC-CHF
Total cholesterol					
(mg/g liver)	6.25 ± 2.96	99.18 ± 17.86 *	75.67 ± 13.08 *^#^	53.29 ± 20.85 *^#+^	58.78 ± 10.29 *^#^
(g/liver)	0.12 ± 0.08	3.53 ± 0.95 *^#^	2.53 ± 0.57 *^#^	1.54 ± 0.67 *^#+^	1.50 ± 0.47 *^#+^
Triglyceride					
(mg/g liver)	33.56 ± 19.07	94.90 ± 18.48 *	107.50 ± 24.17 *	87.77 ± 25.33 *	63.55 ± 21.37 ^+^
(g/liver)	0.65 ± 0.50	3.37 ± 0.68 *	3.69 ± 0.85 *	2.57 ± 1.01 *	1.66 ± 0.74 ^#+^

Results are expressed as the mean ± SD for each group (n = 7). The significant difference (*p* < 0.05) was analyzed by one-way ANOVA. * *p* ˂ 0.05 as compared with the ND group. ^#^ *p* ˂ 0.05 as compared with the HF group. ^+^ *p* ˂ 0.05 as compared with the LC group. ND: normal control diet; HF: high-fat diet; LC: low-carbohydrate + high-fat diet; LC-CH: low-carbohydrate + high-fat diet + 5% chitosan; and LC-CHF: low-carbohydrate + high-fat diet + 5% chitosan + 5% fish oil.

**Table 6:** The results of fecal weight, total cholesterol, and triglyceride concentration in rats fed with the different experimental diets after 15 weeks.

	ND	HF	LC	LC-CH	LC-CHF
Feces wet weight (g/day)	2.57 ± 0.38	2.86± 0.67	3.39 ± 0.35 *	2.80 ± 0.46	2.52 ± 0.51 ^+^
Feces dry weight (g/day)	2.05 ± 0.22	2.18 ± 0.39	2.66 ± 0.33 *	2.14 ± 0.26 ^+^	2.01 ± 0.39 ^+^
Total cholesterol					
(mg/g feces)	2.53 ± 0.79	7.54 ± 1.37 *	11.08 ± 1.24 *^#^	12.08 ± 1.93 *^#^	15.05 ± 2.96 *^#+&^
(mg/day)	5.11 ± 1.33	16.73 ± 5.15 *	29.58 ± 5.44 *^#^	26.03 ± 6.13 *^#^	29.82 ± 6.21 *^#+&^
Triglyceride					
(mg/g feces)	6.30 ± 1.49	9.31 ± 2.62	9.95 ± 2.58 *	8.90 ± 1.70	7.17 ± 0.77
(mg/day)	12.80 ± 2.47	21.01 ± 9.07	26.40 ± 7.30 *	18.97 ± 3.72	14.43 ± 3.22

Results are expressed as the mean ± SD for each group (n = 7). The significant difference (*p* < 0.05) was analyzed by one-way ANOVA. * *p* ˂ 0.05 as compared with the ND group. ^#^ *p* ˂ 0.05 as compared with the HF group. ^+^ *p* ˂ 0.05 as compared with the LC group. ^&^ *p* ˂ 0.05 as compared with the LC-CH group. ND: normal control diet; HF: high-fat diet; LC: low-carbohydrate + high-fat diet; LC-CH: low-carbohydrate + high-fat diet + 5% chitosan; and LC-CHF: low-carbohydrate + high-fat diet + 5% chitosan + 5% fish oil.

## Data Availability

The data presented in this study are available from the corresponding author upon reasonable request. The data are not publicly available due to privacy policy.
